# Uncovering the Genetic Basis of Porcine Resilience Through GWAS of Feed Intake Data

**DOI:** 10.3390/ani15223269

**Published:** 2025-11-12

**Authors:** Zhenyu Wang, Wenshui Xin, Mengyu Li, Dongdong Duan, Jinyi Han, Mingyu Wang, Shenping Zhou, Xinjian Li

**Affiliations:** Sanya Institute, Hainan Academy of Agricultural Sciences, Sanya 572025, China; wzyhan2017@163.com (Z.W.); xinwenshui@yeah.net (W.X.); mengyuli2020@163.com (M.L.); duandongd@126.com (D.D.); hjy7jane@126.com (J.H.); wangmingyu@nwafu.edu.cn (M.W.)

**Keywords:** pig, feed intake, resilience trait, GWAS, candidate gene

## Abstract

Resilience usually refers to the ability of an animal to be minimally affected by disturbance or to quickly return to its pre-disturbance state. Pigs with strong resilience usually have better production performance and higher tolerance to common diseases. This study utilized feed intake records collected by a Feed Intake Recording Equipment from three pig breeds (Duroc: 823; Landrace: 582; and Yorkshire: 2032). Six resilience traits were constructed using the root mean square error (RMSE) of daily feed intake and feeding duration, cumulative feed intake, and cumulative feeding duration derived from ordinary least squares (OLS) regression, along with quantile regression (QR) estimates of daily feed intake and feeding duration. The correlations between these resilience traits and production traits were subsequently estimated. Single-trait genome-wide association studies (GWASs) were performed using imputed resequencing data to identify key genomic regions and candidate genes associated with resilience traits. The estimated heritability ranged from 0.103 to 0.267 for resilience traits and 0.293 to 0.560 for production traits. Moderate genetic correlations were observed among the resilience traits, while moderate to high genetic correlations were found between resilience traits and production traits. In particular, the traits RMSE_FI_, RMSE_CFI_, QR_FI_, and QR_FD_ exhibited significant moderate to high correlations with most production traits. A genome-wide association study (GWAS) based on imputed whole-genome sequencing data was conducted to identify genomic regions associated with resilience traits in pigs. Using single-trait mixed linear models, 188 SNPs were identified and annotated to 44 candidate genes. Several of these genes (*CD74*, *CSF1R*, and *HTR4*) are involved in host immune responses and signal transduction pathways. These findings contribute to a better understanding of the genetic mechanisms underlying resilience in pigs and provide insights for enhancing genomic selection in pig breeding.

## Simple Summary

Recent climatic variability driven by global warming has introduced new challenges for livestock production, motivating the swine industry to breed animals with enhanced adaptability to extreme environments. Using daily feed-intake records collected by automated feeding systems, we estimated the genetic parameters of porcine resilience traits. A genome-wide association study further identified candidate genes associated with resilience, including *CD74*, *CSF1R*, and *HTR4*. These findings provide a biological basis for understanding resilience in livestock and inform genomic selection strategies.

## 1. Introduction

Pork ranks among the most widely consumed meats worldwide, representing roughly one-third of global meat production [1]. Over recent decades, intensive genetic selection has markedly enhanced domestic pigs’ growth rate, carcass characteristics, and reproductive performance [2,3]. However, the improvement in production performance did not lead to an increase in disease resistance. When pigs are infected with diseases or subjected to unfavorable production conditions, their production performance may be negatively impacted [4,5]. Resilience refers to an animal’s capacity to minimize the impact of disturbances or to rapidly return to its pre-disturbance state [6,7,8]. Highly resilient individuals not only recover more quickly from stressors but also tend to possess stronger immune systems that better resist common diseases [6,9]. Consequently, improving resilience in domestic pigs has become an increasingly important objective in breeding programs.

However, defining and measuring resilience traits presents significant challenges. Recent studies suggest that a promising approach involves analyzing the variability in longitudinally recorded data as a quantitative indicator of resilience [6,8,10]. This method has been successfully applied to various livestock production traits—for instance, daily milk yield in dairy cattle [11,12], egg production in laying hens [13], and body weight in meat sheep [14]. In pigs, Putz et al. [15] employed a natural disease challenge model to assess disease resistance during the weaning-to-finishing period. They found that the root mean square error (RMSE) and quantile regression (QR) of feed intake (FI) and feeding duration (FD) were highly correlated with mortality and recovery rates. Another study reported that resilience indicators derived from deviations in FI were favorably associated with feed efficiency traits, and that offspring with higher resilience showed a 2.5% increase in survival rate [16]. Moreover, several other studies have shown that resilience indicators based on deviations in FI and FD exhibit moderate heritability and moderate-to-high genetic correlations with traits such as feed efficiency, feeding behavior, growth, and animal welfare [7,17,18]. As a result, FI and FD are considered among the most promising indicators for developing resilience traits in livestock.

Genome-wide association studies (GWAS) are widely recognized as an effective tool for dissecting the genetic architecture of complex traits and accelerating genetic improvement in pigs [19,20]. Previous studies have identified several genomic regions associated with immune resilience in pigs. For instance, Chen et al. [21] conducted a GWAS on natural antibody levels in Yorkshire × Landrace pigs and proposed *CD14* and *MIF*, among others, as candidate genes for disease resilience. In a large-scale experimental infection study with Porcine circovirus 2 (PCV2), Walker et al. identified *SYNGR2* as a strong candidate gene, demonstrating that its mutation affects PCV2 replication [22]. Furthermore, Li et al. [23] identified significant immune-related QTL regions and candidate genes in a Duroc population based on resilience indicators derived from FI and FD. Similarly, Casto-Rebollo et al. [16] detected overlapping genomic regions for resilience indicators and feed conversion efficiency in Pietrain pigs, suggesting potential roles for candidate genes in immune response regulation and/or metabolism. However, research on resilience indicators in commercial pig breeds remains limited.

In this study, we collected feed-intake records from 3437 pigs with the objectives of (1) estimating the heritabilities and genetic correlations among six resilience traits, (2) assessing the relationships between resilience traits and production traits, and (3) performing single-trait GWAS using mixed linear models to identify genomic regions associated with resilience. Our findings will improve understanding of the genetic architecture underlying porcine resilience and provide a theoretical foundation for molecular breeding in pigs.

## 2. Materials and Methods

### 2.1. Animals and Data Collection

The study was carried out on Duroc, Landrace, and Yorkshire pigs from a commercial company in Henan Province, China. The nucleus pig test barn consisted of 17 compartments with 6 pens per compartment and on average 15 pigs per pen (1.7 m^2^ per pig). Water was provided ad libitum in each pen from one nipple drinker and feed was provided with a Feed Intake Recording Equipment (FIRE^®^) system (Osborne Industries, Inc., Osborne, KS, USA). The FIRE system recorded the following data for each animal visit to the testing station: individual ID, visit start and end times (accurate to 1 s), initial and final trough weights (accurate to 1 g), and body weight (accurate to 0.1 kg). Before data quality control (QC), the dataset comprised 4102 pigs born between December 2023 and April 2025. In total, these pigs had 3,119,697 FIRE recordings for weight, feed intake and feeding duration. Furthermore, pedigrees can be traced back fiver generations.

### 2.2. Quality Control

This study investigates variability in longitudinal data from FIRE, and links this variability with underlying biological genetic factors. Therefore, it is vital that variability due to technical errors and/or noise are removed as much as possible (Supplementary File S1: Table S1). Individual feed intake visits were processed and cleaned using the methods of Casey et al. [24] and were aggregated into daily totals for each pig, including total amount of feed consumed (kg) and duration (time) at the feeder (minutes). Animals with fewer than 60 recorded entries were excluded from the analysis. Instances where the daily feed intake, or daily feeding duration exceeded the mean by more than four standard deviations were treated as missing values. These missing values were estimated using a 5-day rolling average. If two consecutive periods had missing values, the original value was retained [23]. Next, only FIRE records within the age range of 90 to 150 days were retained to standardize the age of the animals. This range was chosen because the majority of our FIRE records fall within this window, and most pigs exhibit linear growth during this period (Supplementary File S1: Figure S1). After final quality control, a total of 209,657 FI, FD and weight records from 3437 pigs were retained.

### 2.3. Resilience Traits and Production Traits

**Figure 1:**
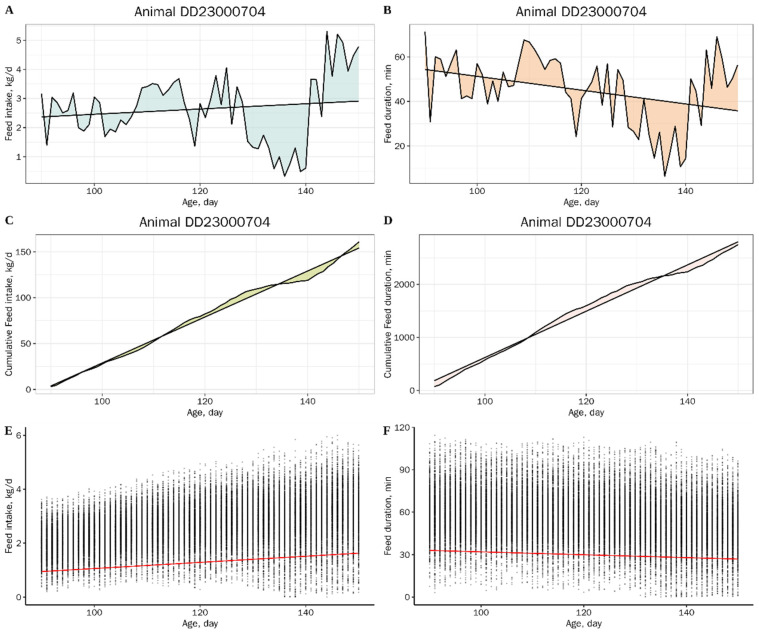
Root mean square errors of feed intake (**A**) and feeding duration (**B**) as examples of resilience traits. Root mean square errors of cumulative feed intake (**C**) and cumulative feeding duration (**D**) as examples of resilience traits. Duration is the residence time in minutes. Each animal was given a record of changes in feed intake or duration with age. Quantile regression (QR) plots of daily feed intake (**E**) and feeding duration (**F**). Each point represents an observation of a single animal. The red line represents 5% of QR.

To quantify individual resilience, we constructed three groups of resilience traits based on FI and FD data. In the first group, resilience was measured by calculating the RMSE from OLS linear regressions of FI and FD against age, denoted as RMSE_FI_ and RMSE_FD_ (Figure 1A,B). Higher RMSE values indicate lower resilience. In the second group, we employed QR to construct population-level 5th percentile regression lines. Instances where an individual’s daily FI or FD fell below this threshold were defined as fasting days, and the proportion of fasting days per individual was calculated (Figure 1E,F). A higher proportion of fasting days indicates lower resilience. Previous studies have reported that during periods of illness, FI often approaches zero, resulting in a flattened cumulative feed intake (CFI) curve. This fluctuation in cumulative feeding behavior can also reflect an animal’s resilience [25,26]. Therefore, in the third group, we calculated the RMSE of both CFI and Cumulative feed duration (CFD) curves to evaluate individual resilience. As with the other groups, higher RMSE values reflect lower resilience (Figure 1C,D).

To compare production performance across individuals, we evaluated seven traits encompassing growth, feeding behavior, and feed efficiency. For growth traits, average daily gain (ADG) between 90 and 150 days of age was calculated as:

$$\mathrm{ADG}\left(kg/d\right)=\frac{weight\left(kg\right)\;at\;maximum\;age\;-\;weight\left(kg\right)\;at\;minimum\;age}{maximum\;age\left(d\right)\;-\;minimum\;age\left(d\right)}$$

The adjusted age and adjusted backfat thickness at 100 kg body weight were estimated using the following equations:

$$adjusted\;100\;kg\;AGE\;=\;Measured\;age+\;\frac{\left(Measured\;body\;weight\;-\;100\;kg)(Measured\;age\;-\;A\right)}{Measured\;body\;weight}$$

$$adjusted\;100\;kg\;BF\;=\;Measured\;bf\;+\;\frac{\left(Measured\;body\;weight\;-\;100\;kg)(Measured\;bf\right)}{Measured\;body\;weight\;-\;B}$$

where *A* and *B* are breed- and sex-specific correction coefficients provided in Supplementary File S1: Table S2.

For feeding behavior traits, we computed average daily feed intake (ADFI, kg) and average daily feeding duration (ADFD, minutes) to assess feeding performance during the 90–150 days period. For feed efficiency traits, we calculated the feed conversion ratio (FCR) as:

$$FCR=\frac{ADFI}{ADG}$$

In addition, residual feed intake (RFI) was calculated using a multiple regression model [27]:

$$RFI=ADFI-(b_{1}\times ADG+b_{2}\times MBW)$$

where MBW is the metabolic BW (mid-test BW^0.75^), and *b_1_* and *b_2_* are the partial regression coefficients of ADFI on ADG and MBW, respectively.

### 2.4. Statistical Analysis

To estimate the genetic parameters of the resilience trait and the production trait, we used the single-trait and two-trait animal models of the HIBLUP v1.53 [28] software for evaluation. The models are shown as follows:

$$y_{ijklmno}=\mu_{i}+{Pen\_batch}_{j}+{breed}_{k}+{sex}_{l}+a_{m}+{litter}_{n}+e_{ijklmno}$$

where $y_{ijklmn}$ are the phenotypic records of resilience and production traits; $\mu_{i}$ is the total mean for trait i; ${Pen\_batch}_{j}$ is the fixed effect of the pen × batch j (278 levels); ${breed}_{k}$ is the fixed effect of breed j (3 levels); ${sex}_{l}$ is the fixed effect of sex l (2 levels); $a_{m}$ is the random additive genetic effect; ${litter}_{n}$ is the random effect of litter effect n; and $\varepsilon_{ijklmn}$ is the random residual effects. For the above model, the heritability was defined as $h^{2}=\frac{\sigma_{a}^{2}}{\sigma_{p}^{2}}$, and $\sigma_{p}^{2}=\sigma_{a}^{2}+\sigma_{litter}^{2}+\sigma_{e}^{2}$. The genetic correlation coefficient was calculated as $r=\frac{\sigma_{ij}}{\sqrt{\sigma_{i}^{2}\sigma_{j}^{2}}}$.

### 2.5. Genotype Data

In this study, genomic data were derived from two sources. The first dataset consisted of whole-genome resequencing (WGS) data from 1138 pigs, with an average sequencing depth of 10×. The single nucleotide polymorphism (SNP) calling [29,30,31,32] procedure and chromosomal distribution of the variants are presented in Supplementary File S1. The second dataset involved 856 pigs genotyped using the Porcine 50K SNP Bead Chip (Kangpu Sen Agricultural Technology Co., Ltd., Beijing, China), which included 57,466 genome-wide SNP markers. Plink v1.90 [33] software (Cambridge, MA, USA) to exclude markers not meeting the following criteria: (1) individual genotype call rate below 95%, (2) SNP genotype call rate below 90%, (3) minor allele frequency (MAF) > 0.05, and (4) deviations from Hardy–Weinberg equilibrium (*p* < 10^−6^). SNPs located on sex chromosomes and unplaced genomic regions were excluded from the study. To obtain a higher-resolution dataset, we inputted genotypes from the 50K SNP chip to the whole-genome sequence level using a reference panel of WGS data from 1138 pigs representing three breeds. Imputation was performed with Beagle v5.4 software [34] and achieved an average accuracy (R^2^) of 0.923. Following imputation, SNPs underwent an additional round of quality control. Only high-quality SNPs were retained for downstream analysis. In total, 1616 individuals with both phenotype and genotype data were included in the final dataset, comprising 12,848,070 SNPs for subsequent analyses.

### 2.6. Population Structure Analysis

Population stratification vastly affects GWAS reliability, so software R v4.3.2 and GCTA v1.94.3 were used to evaluate the population structure of three pig populations. The Q–Q plot is a commonly used tool for scanning population stratification in GWAS. In this study, the Q–Q plot was constructed by R v4.3.2. software [35]. Given that the experimental animals in this study originated from three groups, we employed PCA to assess the genetic background similarities among Duroc, Landrace, and Yorkshire pigs. PCA was generated by software GCTA v1.94.3 [36].

### 2.7. Single-Population GWAS

GEMMA v0.98.5 software [37] was applied to a univariate linear mixed model to execute GWAS, and the single-population analysis of the pig populations used the same univariate linear mixed model. Before GWAS, the genomic relatedness matrix (GRM) between individuals was estimated by GEMMA. The matrix form was used in the following statistical model:

y=Wα+Xβ+u+ε

where *y* refers to a vector of phenotypic values for all animals; *W* denotes the incidence matrices of covariates (fixed effects), including birth year and season, breeds, and the top five eigenvectors of PCA; *α* represents the vector of corresponding coefficients with the intercept; *X* corresponds to the vector of marker genotypes; *β* specifies the corresponding effect size of the marker and is an estimate of the maker/SNP additive effect; u stands for the vector of random effects with $u\sim {MVN}_{n}(0,\lambda \tau^{-1}K)$; *ε* is the vector of random residuals with $\varepsilon \sim {MVN}_{n}(0,\tau^{-1}I_{n})$; λ signifies the ratio between two variance components; $\tau^{-1}$ is the variance of the residual errors; K is a known *n* × *n* relatedness matrix and $I_{n}$ is an *n* × *n* identity matrix; ${MVN}_{n}$ denotes the *n*-dimensional multivariate normal distribution. Because the Bonferroni correction is overly stringent and reduces statistical power [38], we used the false discovery rate (FDR) to define significance thresholds for single-trait GWAS. FDR was set to 0.001, and the threshold *p*-value was calculated as [39]:

$$P=FDR\times \frac{n}{m}$$

where *n* is the number of resulting *P*-values less than 0.001 and m is the total number of SNPs.

### 2.8. Bioinformatics Analysis

Annotation of genes nearest to significant SNPs was performed using the Variant Effect Predictor (VEP) module from the Ensembl database (http://ensembl.org/Sus_scrofa/Info/Index (accessed on 25 June 2025), Genome assembly: Sscrofa11.1). To explore candidate genes involved in pathways and biological processes, KEGG (Kyoto Encyclopedia of Genes and Genomes) and GO (Gene Ontology) analyses were conducted using KOBAS 3.0 (http://bioinfo.org/kobas (accessed on 8 July 2025)) [40]. To enhance the reliability of gene function annotation, a more comprehensive and well-annotated human database was used as the reference for functional enrichment analysis. The significance of enriched pathways was assessed with Fisher’s exact test, with a threshold of *p* < 0.05.

## 3. Results

### 3.1. Phenotype Statistics

This study analyzed the phenotypes of six resilience traits and seven other traits. After quality control filtering, a total of 3437 animals were included in the subsequent analysis. As shown in Table 1, the mean values of the six resilience traits were 0.56, 11.38, 3.55, 41.84, 5.00 and 5.00, respectively. Notably, both QRFI and QRFD had minimum values of zero, which is attributed to some animals not experiencing any days below the 5% threshold. Table 1 also presents the descriptive statistics for the resilience traits, feeding behavior traits, and production traits. Heritability for each trait was estimated based on pedigree information: resilience traits ranged from 0.103 to 0.267, growth traits ranged from 0.293 to 0.560, feeding behavior traits ranged from 0.324 to 0.342, and feed efficiency traits ranged from 0.324 to 0.343.

### 3.2. Correlation

To assess correlations between resilience traits, we estimated both phenotypic and genetic correlations among the traits. Table 2 displays phenotypic correlations ranging from −0.035 to 0.588 and genetic correlations ranging from −0.426 to 0.823 among resilience-related traits. A strong positive correlation was observed between RMSE_FI_ and RMSE_FD_, indicating that pigs with higher variability in daily feed intake also exhibited higher variability in feeding duration. Notably, QR_FD_ showed moderately negative genetic correlations with RMSE_FI_, RMSE_FD_, RMSE_CFI_, and RMSE_CFD_. This suggests that pigs genetically predisposed to more days without feeding exhibited higher genetic variability in feed intake and feeding duration.

**Figure 2:**
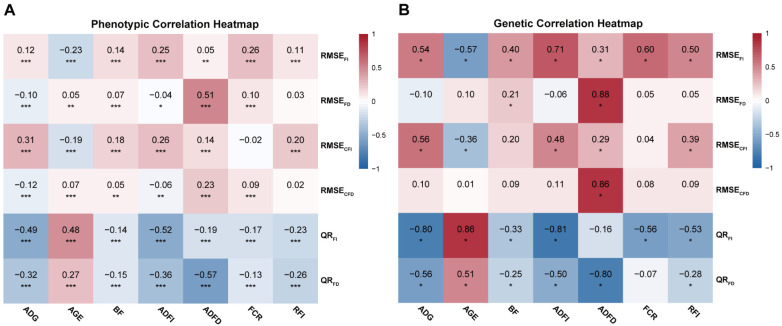
Estimates of phenotypic (r_p_) (**A**) and genetic correlations (r_g_) (**B**) between resilience-related traits and other traits. Abbreviations: RMSE, root mean square error; QR, quantile regression. RMSE_FI_, RMSE of daily feed intake; RMSE_FD_, RMSE of daily feed duration; RMSE_CFI_, RMSE of cumulative feed intake; RMSE_CFD_, RMSE of cumulative feed duration; QR_FI_, Quantile regression of daily feed intake; QR_FD_, Quantile regression of daily feed duration; ADG, Average daily gain from 90 to 150 d; AGE, Adjust 100 kg age; BF, Adjust 100 kg backfat thickness; ADFI, Average occupation time in feeder per day; ADFD, Average number of visits to feeder per day; FCR, Feed conversion ratio; RFI, Residual feed intake. *: *p* < 0.05, **: *p* < 0.01, ***: *p* < 0.001.

To understand the relationship between resilience traits and productive traits, we also estimated the phenotypic and genetic correlations between resilience traits and productive traits. Figure 2 shows that the phenotypic correlation range between resilience related traits and feeding behavior or production traits is from −0.57 to 0.51, and the genetic correlation range is from −0.81 to 0.88. Both RMSE_FI_ and RMSE_FD_ showed moderate to strong positive phenotypic correlations with ADFI and ADFD (e.g., RMSE_FI_ vs. ADFI: 0.27), indicating that animals with greater fluctuations in feed intake or feeding time tended to consume more feed and spend more time eating. The genetic correlations between resilience traits and feeding behavior/production traits ranged from −0.80 (QR_FI_ vs. ADG) to 0.88 (RMSE_FD_ vs. ADFD). In addition, QR_FI_ and QR_FD_ were moderately negatively correlated phenotypically with average daily gain and age at 100 kg body weight, and exhibited significantly negative genetic correlations with ADG (−0.80 and −0.56, respectively), suggesting that a higher number of fasting days has an adverse effect on pig growth.

### 3.3. Population Genetic Structure and GWAS Results

The animals in this study were sourced from three different pig breeds. Principal component analysis (PCA) was conducted to identify potential population stratification (Supplementary File S1: Figure S2). The PCA plot shows that Duroc, Landrace, and Yorkshire pigs formed three distinct clusters, indicating that the three populations have relatively independent genetic backgrounds. Additionally, to assess the presence of potential false positive signals in the GWAS results, we calculated the genomic inflation factor (λ). The λ values for each population ranged from 0.987 to 1.047, and the Q-Q plots showed no signs of inflation, indicating no population stratification and that the GWAS results are reliable (Supplementary File S1: Figure S3).

**Figure 3:**
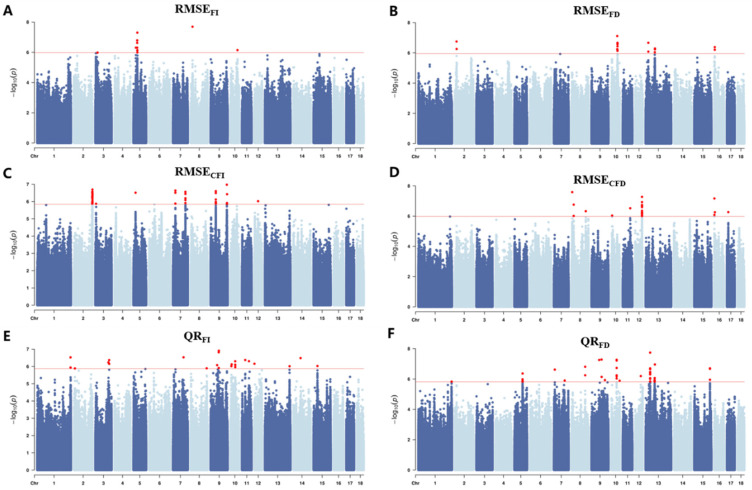
Manhattan plots of GWAS for resilience traits in pig populations In the Manhattan plots, the red solid line denotes the significance threshold, and red dots indicate significant SNPs. Manhattan plot for (**A**) RMSE_FI_, (**B**) RMSE_FD_, (**C**) QR_FI_, (**D**) QR_FD_, (**E**) RMSE_CFI_, (**F**) RMSE_CFD_.

A total of 188 SNPs were identified as significantly associated with resilience traits in this study (Table 3 and Figure 3). Among them, 11 SNPs were linked to RMSE_FI_ and located on SSC 3, 5, 8, and 10; 25 SNPs were associated with RMSE_FD_ and distributed on SSC 2, 10, 13, and 16; 25 SNPs correlated with QR_FI_ were found on SSC 1, 2, 3, 7, 8, 9,10, 11, 12, 13, 14, and 15; 44 SNPs related to QR_FD_ were located on SSC 1, 5, 7, 8, 9, 10, 12, 13, and 15; 57 SNPs associated with RMSE_CFI_ were distributed on SSC 2, 3, 5, 7, 9, and 12; and 33 SNPs connected to RMSE_CFD_ were situated on SSC 8, 10, 11, 12, 16, and 17. Variant annotation using the Variant Effect Predictor identified 44 candidate genes corresponding to these significant loci (Supplementary File S2). Table 4 includes only strong candidate genes that are biologically relevant to resilience traits-for example, genes known to be involved in immune regulation, stress responses, or metabolic adaptation.

### 3.4. Functional Annotation

We performed GO term and KEGG pathway enrichment analyses for all candidate genes to identify their roles within established metabolic pathways. Based on the functional annotation results, several pathways associated with porcine resilience were identified. These include multiple KEGG pathways such as Cell adhesion molecules (CAMs), Wnt signaling pathway, and HIF-1 signaling pathway. In addition, enriched GO terms include positive regulation of kinase activity, CD40 signaling pathway, and regulation of feeding behavior, MHC class II protein complex.

## 4. Discussion

In recent years, the drastic climatic fluctuations driven by global warming have posed new challenges to livestock production [41]. Enhancing environmental adaptability or resilience has consequently become one of the emerging focuses in pig breeding. In earlier studies, resilience was typically assessed using immune-related indicators. For instance, Chen et al. [21] reported heritability estimates ranging from 0.12 to 0.24 for natural antibodies and total IgG levels in piglet blood. Similarly, Dervishi et al. [42] estimated heritability values between 0.11 and 0.39 when using plasma metabolites as potential genetic indicators. The post-vaccination increase in the acute-phase protein haptoglobin (HP) also showed moderate to low heritability (0.16) [43]. Moreover, deviations of individual growth curves from expected body weight have been proposed as potential indicators of resilience. Consequently, continuous longitudinal phenotypes—such as fluctuations in feed intake during the growth period—appear to be superior indicators for assessing resilience and may be effectively incorporated into breeding programs. Putz et al. [15] employed a natural disease model from weaning to finishing and demonstrated that daily variations in feed intake or feed delivery could serve as measures of resilience, with moderate heritability estimates (0.15–0.26). Kavlak et al. [17] reported low (0.08 ± 0.04, CV_FI_) to moderate (0.23 ± 0.05, CV_FD_) estimates, while Homma et al. [18] obtained moderate estimates (0.31 ± 0.03 for LnCV_FI_ and 0.36 ± 0.03 for LnCV_FD_). In addition, Gorssen et al. [7] found both relatively low (0.09 ± 0.02, QRFI) and moderate (0.28 ± 0.03, LnMSE_FD_) heritability estimates. In the present study, we analyzed feed intake records from 3437 pigs and, following the approach of Putz et al. [15], constructed six resilience indicators based on daily feed intake data to estimate their genetic parameters. The estimated heritabilities ranged from low (0.103 ± 0.04) to moderate (0.267 ± 0.06), which aligns well with previous reports. Overall, these findings confirm that evaluating pig resilience using feed intake data is a practical and reliable approach for genetic improvement programs.

In addition, we estimated the correlations between resilience traits and production traits (Figure 2). The results showed that both QR_FI_ and QR_FD_ exhibited strong negative correlations with most production traits, except for AGE. Similarly, Kavlak et al. [17] conducted a genetic analysis of resilience indicators based on FI and FD in Finnish Landrace pigs and reported negative genetic correlations of QR_FI_ and QR_FD_ with traits such as ADG and FCR. These findings are consistent with our results. This suggests that pigs with a higher proportion of fasting days tend to have lower resilience and poorer production performance. In future studies, we plan to collect additional phenotypic data to further validate the impact of these two traits on production efficiency.

Genome-wide association studies (GWAS) have become a powerful approach for uncovering causal variants underlying complex traits. Tong et al. [44] reported that imputing genotypes to the whole-genome sequence (WGS) level can reveal novel and stronger GWAS signals. Therefore, in the present study, we imputed the collected 50K SNP genotypes to WGS-level data to enhance detection power and identify key mutations associated with resilience traits. A total of 179 significant SNPs were identified and annotated to 44 candidate genes using the VEP. Among these, *STX5*, *HTR4*, *CSF1R*, *TCOF1*, *CD74* and *TF* were considered strong candidate genes.

In this study, a key QTL associated with resilience traits was identified on SSC2, spanning 149.29–151.10 Mb. Within this region, 31 SNPs were detected, and annotation using the VEP revealed four candidate genes: *HTR4, CSF1R, TCOF1*, and *CD74* (Table 4). Notably, two significant SNPs were located in the intronic region of the *CD74* gene. GO analysis showed that *CD74* is involved in the formation and trafficking of *MHC* class II peptide complexes, which are essential for eliciting CD4^+^ T-cell responses [45]. On the cell surface, *CD74* acts as a receptor for the cytokine macrophage migration inhibitory factor (MIF) in various cell types [46]. In immune cells, the binding of MIF to *CD74* initiates downstream signaling cascades that regulate cell proliferation and survival [47]. Previous studies have also implicated *CD74* and *CD81* in defining subsets of innate immune cells. One significant SNP was located within an intronic region of the *CSF1R* gene (colony-stimulating factor 1 receptor). This gene is activated by its ligands, colony-stimulating factor 1 (*CSF-1*) and interleukin-34 (IL-34) [48]. The *CSF1R* signaling pathway often interacts with other cytokine and receptor signaling cascades, such as IFN-γ, GM-CSF, TLR, and IL-4/IL-13 pathways, collectively regulating macrophage polarization, functional state, and the balance between survival and apoptosis [49,50]. In addition, previous studies have suggested that polymorphisms in *CSF1R* may be associated with the severity of APC(1311/+) porcine polyposis [51]. Another candidate gene within this QTL is *HTR4*, which encodes one of the 5-hydroxytryptamine (5-HT) receptors. Binding of 5-HT to *HTR3*, *HTR4*, and *HTR7* has been reported to upregulate the pro-inflammatory cytokine IL-6, suggesting a role for *HTR4* in immune regulation and inflammatory response [52]. Additionally, one SNP located within the intronic region of the *STX5* gene on SSC2 was identified. The *STX5* gene has previously been associated with climatic resilience (CR) in pigs, further supporting its potential role in adaptation-related traits [53].

**Figure 4:**
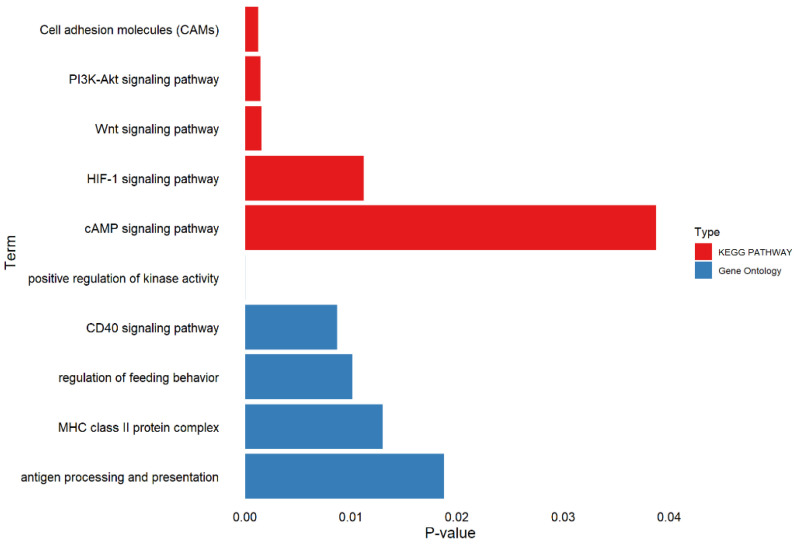
Bar plot illustrating the *p*-values for selected terms related to resilience trait.

On SSC13, a SNP was identified within an intronic region of the *TF* (transferrin) gene (Table 4). It has been reported that transferrin-mediated iron provision is essential for productive infections by many bacterial pathogens, while iron sequestration by transferrin represents a first-line defense against bacterial invasion [54]. KEGG enrichment analysis revealed that *TF* is involved in the HIF-1 signaling pathway (Figure 4). This pathway, mediated by hypoxia-inducible factor 1 (HIF-1), plays a vital role in cellular responses to low oxygen availability. HIF-1 is a transcription factor composed of two subunits: an oxygen-regulated α subunit and a constitutively expressed β subunit, both conserved across all metazoans. The HIF-1 pathway is crucial for sensing hypoxia-induced metabolic shifts, regulating cell proliferation, and initiating inflammatory responses [55]. These findings suggest that *TF* should be regarded as a powerful candidate gene for the elastic trait.

Overall, compared with previous studies, the present research utilized imputed whole-genome sequence data and a larger sample size, thereby improving the power to detect significant loci. However, several limitations should be noted. Most significant variants were located in non-coding regions, which complicated the identification and validation of causal mutations. Furthermore, the limited number of animals restricted breed-specific analyses. Therefore, future work will expand the sample size and integrate multi-omics approaches to further investigate causal variants underlying resilience-related traits.

## 5. Conclusions

In this study, we derived six resilience traits based on daily feed intake and feeding duration data, using root mean square error and quantile regression methods. Among them, RMSE_FI_, RMSE_FD_, RMSE_CFI_, QR_FI_, and QR_FD_ exhibited moderate heritability, while RMSE_CFD_ showed relatively low heritability. Interestingly, QR_FI_ and QR_FD_ displayed consistently negative correlations with most production traits, indicating that pigs with lower resilience tend to have poorer production performance. Furthermore, the GWAS analysis of resilience traits identified genomic regions associated with immune response and adaptability, highlighting their potential biological relevance. These findings have important implications for improving animal health and enhancing production efficiency. Nevertheless, further research is needed to validate these results. In particular, it is essential to investigate the functional roles of the identified candidate genes in resilience and to integrate these resilience indicators into future breeding programs.

## Figures and Tables

**Table 1:** Descriptive statistics for analyzed traits in Yorkshire, Landrace, and Duroc pigs.

Trait	Abbreviations ^1^	Unit	N	Mean	SD	Min	Max	h^2^ (SE)
**Resilience trait**								
RMSE of daily feed intake	RMSE_FI_		3437	0.56	0.15	0.18	1.29	0.237 (0.05)
RMSE of daily feed duration	RMSE_FD_		3437	11.38	3.05	4.43	26.30	0.267 (0.06)
RMSE of cumulative feed intake	RMSE_CFI_		3437	3.55	1.29	0.80	8.92	0.230 (0.05)
RMSE of cumulative feed duration	RMSE_CFD_		3437	41.84	22.71	5.69	182.85	0.103 (0.04)
Quantile regression of daily feed intake	QR_FI_		3437	5.00	6.19	0	77.05	0.160 (0.04)
Quantile regression of daily feed duration	QR_FD_		3437	5.00	6.06	0	59.02	0.171 (0.04)
**Growth trait**								
Average daily gain from 90 to 150 d	ADG	kg	3437	1.05	0.14	0.50	1.54	0.293 (0.06)
Adjust 100 kg age	AGE	day	3295	143.97	9.73	119.91	180.28	0.357 (0.06)
Adjust 100 kg backfat thickness	BF	mm	3293	10.75	2.06	5.22	22.20	0.560 (0.07)
**Feeding behaviour traits**								
Average occupation time in feeder per day	ADFI	kg	3437	2.51	0.39	1.12	4.02	0.342 (0.06)
Average number of visits to feeder per day	ADFD	min	3437	55.94	9.62	29.23	97.3	0.324 (0.06)
**Feed efficiency trait**								
Feed conversion ratio	FCR		3437	2.40	0.27	1.24	3.95	0.324 (0.061)
Residual feed intake	RFI	kg	3437	0	0.23	−1.14	0.88	0.343 (0.061)

^1^ RMSE, root mean square error; QR, quantile regression. RMSE_FI_, RMSE of daily feed intake; RMSE_FD_, RMSE of daily feed duration; RMSE_CFI_, RMSE of cumulative feed intake; RMSE_CFD_, RMSE of cumulative feed duration; QR_FI_, Quantile regression of daily feed intake; QR_FD_, Quantile regression of daily feed duration; The greater the value of the above six traits, the smaller the resilience. ADG, Average daily gain from 90 to 150 d; AGE, Adjust 100 kg age; BF, Adjust 100 kg backfat thickness; ADFI, Average occupation time in feeder per day; ADFD, Average number of visits to feeder per day; FCR, Feed conversion ratio; RFI, Residual feed intake; N represents the number of animals with available data for each trait. For adjusted 100 kg age (AGE) and adjusted 100 kg backfat thickness (BF), some animals lacked actual measurements, resulting in fewer observations than the total population (N = 3437).

**Table 2:** Estimates of phenotypic (below diagonal) and genetic correlations (above diagonal) within resilience-related traits.

Trait	RMSE_FI_ (SE)	RMSE_FD_ (SE)	RMSE_CFI_ (SE)	RMSE_CFD_ (SE)	QR_FI_ (SE)	QR_FD_ (SE)
RMSE_FI_	-	0.521 (0.099) *	0.301 (0.135) *	0.417 (0.167) *	−0.258 (0.152)	−0.030 (0.147)
RMSE_FD_	0.588 (0.014) ***	-	0.216 (0.132)	0.823 (0.101) *	0.345 (0.125) *	−0.347 (0.137) *
RMSE_CFI_	0.119 (0.017) ***	0.106 (0.017) ***	-	0.288 (0.185)	−0.266 (0.145)	−0.288 (0.143) *
RMSE_CFD_	0.308 (0.017) ***	0.477 (0.015) ***	0.030 (0.017)	-	0.179 (0.185)	−0.426 (0.179) *
QR_FI_	0.265 (0.017) ***	0.366 (0.016) ***	0.001 (0.017)	0.273 (0.017) ***	-	0.546 (0.121) *
QR_FD_	0.262 (0.017) ***	0.026 (0.017)	−0.035 (0.017) *	0.091 (0.017) ***	0.510 (0.015) ***	-

RMSE, root mean square error; QR, quantile regression. RMSE_FI_, RMSE of daily feed intake; RMSE_FD_, RMSE of daily feed duration; RMSE_CFI_, RMSE of cumulative feed intake; RMSE_CFD_, RMSE of cumulative feed duration; QR_FI_, Quantile regression of daily feed intake; QR_FD_, Quantile regression of daily feed duration. *: *p* < 0.05, ***: *p* < 0.001.

**Table 3:** Significant SNPs for resilience traits.

Trait	SSC	N	Position (Mb)	Top SNP	
				SNP	*p*-Value
RMSE_FI_	3	1	18.24	3_18244026	1.04 × 10^−6^
	5	8	32.13	5_32134126	4.98 × 10^−8^
	8	1	14.94	8_14938763	2.02 × 10^−8^
	10	1	56.18	10_56176108	7.14 × 10^−7^
RMSE_FD_	2	2	13.78	2_13777555	1.78 × 10^−7^
	10	8	47.61–49.68	10_49631485	2.16 × 10^−7^
	13	5	74.96–76.14	13_22269276	5.17 × 10^−7^
	16	3	3.98	16_3980191	4.18 × 10^−7^
QR_FI_	1	3	270.39	1_270386804	1.16 × 10^−6^
	2	1	8.96	2_8960899	1.30 × 10^−6^
	3	3	106.22–112.56	3_112556064	4.28 × 10^−7^
	7	1	86.34	7_86344231	2.96 × 10^−7^
	8	1	129.80	8_129801413	1.28 × 10^−6^
	9	4	52.97 66.05–66.91	9_66914339	1.22 × 10^−7^
	10	6	6.46–8.93 37.71–38.17	10_37759538	5.01 × 10^−7^
	11	2	26.92	11_26916442	4.23 × 10^−7^
	12	1	0.01	12_10921	6.98 × 10^−7^
	13	1	200.95	13_200945969	9.73 × 10^−7^
	14	1	59.23	14_59225961	3.28 × 10^−7^
	15	1	31.74	15_31744440	9.46 × 10^−7^
QR_FD_	1	1	270.91	1_270916635	1.48 × 10^−6^
	5	5	6.71	5_67126971	4.30 × 10^−7^
	7	2	89.69	7_88693147	1.25 × 10^−6^
	8	2	110.7	8_110707400	1.55 × 10^−7^
	9	4	62.88 82.53	9_82536133	5.18 × 10^−8^
	10	7	42.74	10_42741896	5.34 × 10^−8^
	12	1	45.18	12_45187896	6.38 × 10^−7^
	13	19	37.07–37.09 72.99–75.57	13_37080425	1.81 × 10^−8^
	15	3	127.9	15_127864633	1.98 × 10^−7^
RMSE_CFI_	2	31	149.29–151.10	2_151078512	2.67 × 10^−7^
	3	1	6.2	3_6201890	1.33 × 10^−6^
	5	1	17.66	5_17660757	3.06 × 10^−7^
	7	9	20.27–20.28 101.71–101.73	7_20275252	2.31 × 10^−7^
	9	13	42.35–42.49 131.76–132.83	9_131763229	1.05 × 10^−7^
	12	2	29.58	12_29583861	9.51 × 10^−7^
RMSE_CFD_	8	5	5.46 17.33–17.47 115.67	8_17477285	1.74 × 10^−7^
	10	1	6.07	6073366	9.16 × 10^−7^
	11	1	61.07	61073632	3.00 × 10^−7^
	12	21	55.01–55.08	55.67	5.31 × 10^−8^
	16	4	0.955–0.961 4.92	16_955767	6.79 × 10^−8^
	17	1	11.29	11294396	5.42 × 10^−7^

RMSE, root mean square error; QR, quantile regression. RMSE_FI_, RMSE of daily feed intake; RMSE_FD_, RMSE of daily feed duration; RMSE_CFI_, RMSE of cumulative feed intake; RMSE_CFD_, RMSE of cumulative feed duration; QR_FI_, Quantile regression of daily feed intake; QR_FD_, Quantile regression of daily feed duration.

**Table 4:** Candidate genes for resilience trait.

SSC ^1^	Candidate Genes	Position (Mb) ^2^	N ^3^	Consequence
1	*HMCN2*	270.36–270.52	2	intron_variant
	*QRFP*	270.91	1	upstream_gene_variant
	*FIBCD1*	270.91–270.95	1	intron_variant
2	*STX5*	8.93–8.96	1	intron_variant
	*HTR4*	149.73–149.91	7	intron_variant
	*CSF1R*	151.10–151.14	1	intron_variant
	*TCOF1*	151.36–151.40	2	downstream_gene_variant
	*CD74*	151.40	1	downstream_gene_variant
13	*TF*	74.93–74.97	1	intron_variant
16	*CTNND2*	0.50–1.52	3	intron_variant downstream_gene_variant
16	*FBXL7*	4.78–5.19	1	upstream_gene_variant
17	*IKBKB*	11.28–11.34	1	intron_variant

^1^ Sus scrofa chromosome (SSC); ^2^ Position: range of significant SNP in Ensembl; ^3^ N: number of significant SNPs. *HMCN2:* hemicentin 2; *QRFP*: pyroglutamylated RFamide peptide; *FIBCD1*: fibrinogen C domain containing 1; *STX5*: syntaxin 5; *HTR4*: 5-hydroxytryptamine receptor 4; *CSF1R*: colony stimulating factor 1 receptor; *TCOF1*: treacle ribosome biosis factor 1; *CD74*: CD74 molecule; *TF*: transferrin; *CTNND2*: catenin delta 2; *FBXL7*: F-box and leucine rich repeat protein 7; *IKBKB*: inhibitor of nuclear factor kappa B kinase subunit beta.

## Data Availability

The datasets generated and/or analyzed in this study are not publicly available since the test populations consisted of the nucleus herd of the pig breeding company, but are available from the corresponding author on reasonable request.

## Supplementary Materials

The following supporting information can be downloaded at: https://www.mdpi.com/article/10.3390/ani15223269/s1, Supplementary File S1: Table S1: Summary of Quality Control Filters Applied to Feeding Records; Table S2: Correction parameters in adjusted 100 kg AGE and BF formulas for different varieties; Figure S1: Raw Data statistics; Figure S2: PCA plot of population structure showing the top two principal components; Figure S3: Q–Q plots showing the observed versus expected −log *p*-values for resilience traits; Section S1: The process of calling SNP and the distribution of SNP on chromosome; Figure S4: Distribution density of SNPs on each chromosome. Supplementary File S2: Functional Annotation of Significant Variants and Candidate Genes Identified.

## Abbreviations

The following abbreviations are used in this manuscript:

RMSE	Mean square error roots
OLS	Ordinary least squares
QR	Quantile regression
FI	Daily feed intake
FD	Daily feed duration
CFI	Cumulative feed intake
CFD	Cumulative feed duration
ADG	Average daily gain
AGE	Adjust 100 kg age
BF	Adjust 100 kg backfat thickness
ADFI	Average occupation time in feeder per day
ADFD	Average number of visits to feeder per day
FCR	Feed conversion ratio
RFI	Residual feed intake
GWASs	Genome-wide association studies
