# Supplementary material for: Uncovering the Genetic Basis of Porcine Resilience Through GWAS of Feed Intake Data
Source: Animals (Basel). 2025 Nov 12;15(22):3269. doi: 10.3390/ani15223269 (PMC12649676; doi:10.3390/ani15223269)
Supplement: Supplementary file 1 [file animals-15-03269-s001.zip › Supplementary File S1.pdf]

Table S1. Summary of Quality Control Filters Applied to Feeding Records

Item	Quality Control Condition	Records Removed	Error Rate
1	Single feed intake < −0.02 kg	430	0.000137589
2	Single feed intake > 2 kg	6356	0.002033754
3	Feeding duration = 0 s and single feed intake > 0.02 kg or < −0.02 kg	0	0
4	Feeding duration < 0 s	0	0
5	Feeding duration > 3600 s	235	7.51939E-05
6	Single feed intake between 0–0.05 kg and feeding rate > 0.5 kg/min	0	0
7	Single feed intake > 0.05 kg and feeding rate > 0.35 kg/min	19165	0.0061323
8	Feeding rate = 0 and feeding duration > 500 s	0	0
Total	Records removed	26186	0.84%

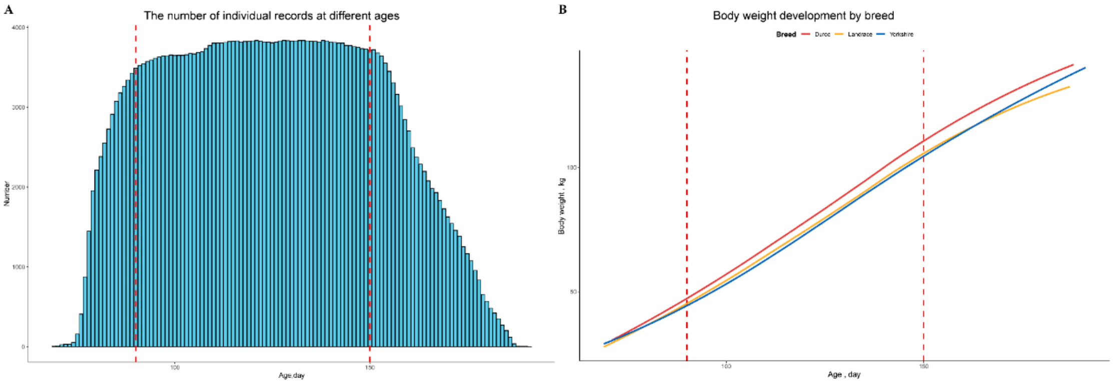

Figure S1. Raw Data statistics. A: The number of individual records at different ages; B: Body weight development by breed.

Table S2. Correction parameters in adjusted 100 kg AGE and BF formulas for different varieties

Breed	A		B	
	Male	Female	Male	Female
Duroc	50.775	46.415	-6.240	-9.440
Landrace	48.441	51.014	-5.623	-3.315
Yorkshire	55.289	49.361	-7.277	-9.440

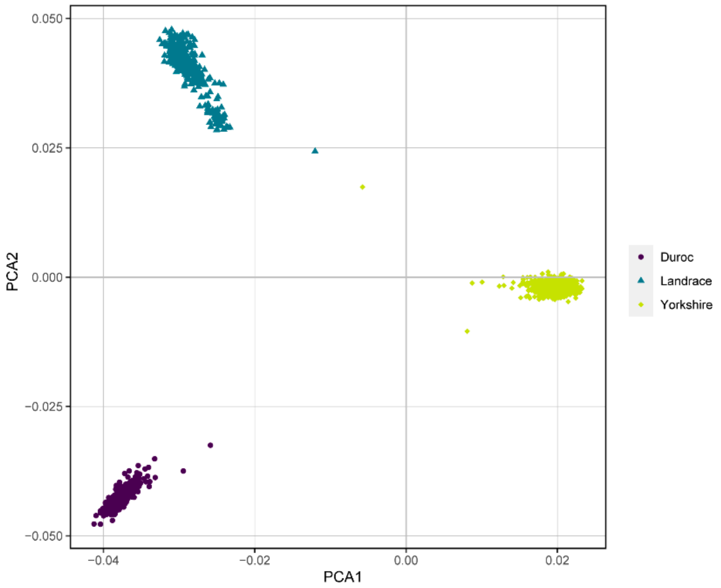

Figure S2. PCA plot of population structure showing the top two principal components. PCA1: principal component 1; PCA2: principal component 2. The purple dot represents the Duroc pigs; the triangle represents the Landrace pigs, and the rhombus represents the Yorkshire pigs.

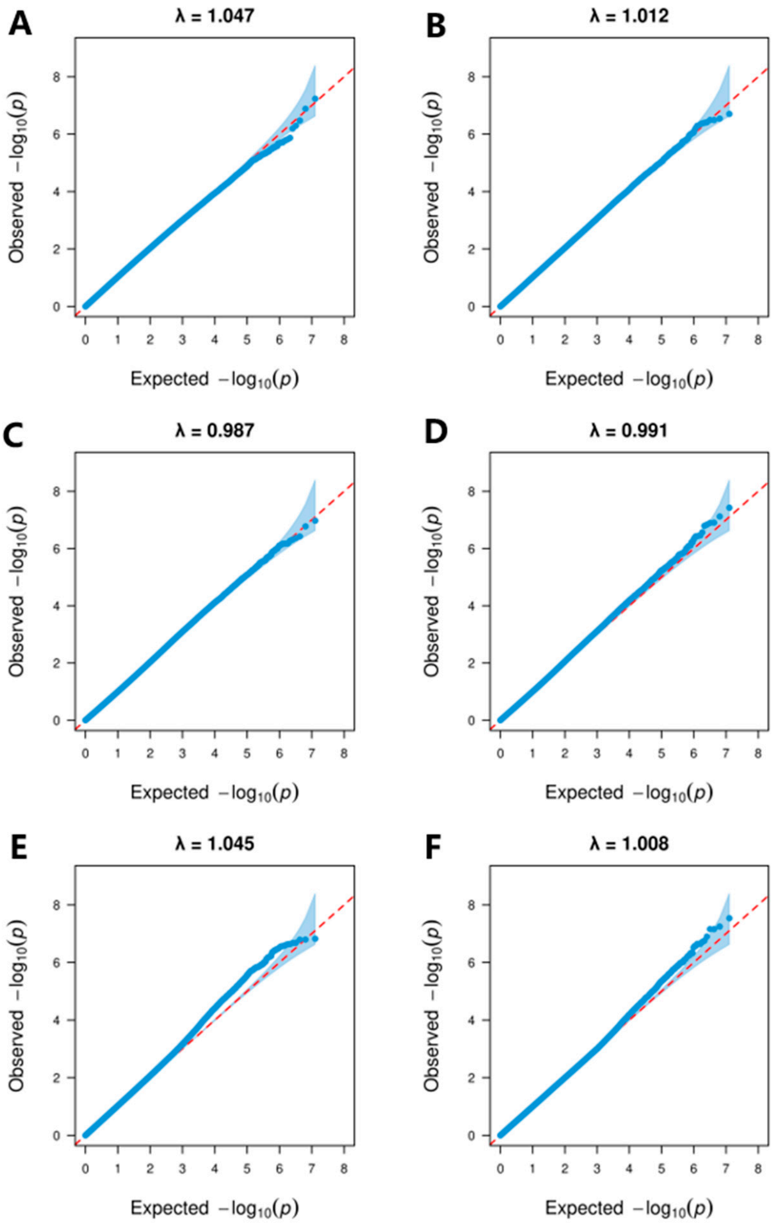

Figure S3. Q–Q plots showing the observed versus expected -log *P*-values for resilience traits. The estimated lambda(*λ*) is shown in the figure. Q–Q plot for (A) $RMSE_{FI}$, (B) $RMSE_{FD}$, (C)QR_{F}, (D) (E) QR_{FD}, $RMSE_{CFII}$, (F) $RMSE_{CFD}$**.**

S1. The process of calling SNP and the distribution of SNP on chromosome

Genomic DNA was extracted from ear tissue using the standard phenol-chloroform method. The quality of the DNA was assessed via UV spectrophotometry (A260/280) and gel electrophoresis. The DNA for each sample was sheared into fragments of 200-800 bp (or 300-400 bp). Next-generation genome sequencing libraries were constructed following the standard protocol of the library preparation kit. Genome sequencing was performed to generate 150 bp (or 125 bp, 100 bp) paired-end reads on the Illumina HiSeq or MGISEQ-2000 platform according to the manufacturer's standard protocols. Using the Fastp v0.20.0 [1], we removed reads with ≥ 10% missing ("N") bases or quality score ≤ 20 for ≥ 50% of bases. All clean reads were aligned to the Sus scrofa reference genome 11.1[2] using BWA v0.7.1770 [3] (BWA-MEM algorithm, default parameters). The sequencing depth and coverage were calculated by Mosdepth v0.3.2 [4]. We sorted the bam files of mapped reads by genome position and marked PCR duplication using Picard v2.21.4 (http://broadinstitute.github.io/picard). The gvcf files were generated using the official pipeline of GATK v4.1.7.0, including program BaseRecalibrator, ApplyBQSR, and HaplotypeCaller. We then jointly genotyped all gvcf files to obtain the final vcf file using GATK GenotypeGVCFs. The variants were filtered with the following criteria: (1) SNP: QD < 2.0, QUAL < 30.0, MQ < 40.0, SOR > 3.0, FS > 60.0, MQRankSum < -12.5, ReadPosRankSum

< -8.0; (2) INDEL: QD < 2.0, QUAL < 30.0, MQ < 40.0, FS > 200.0, ReadPosRankSum < -20.0.

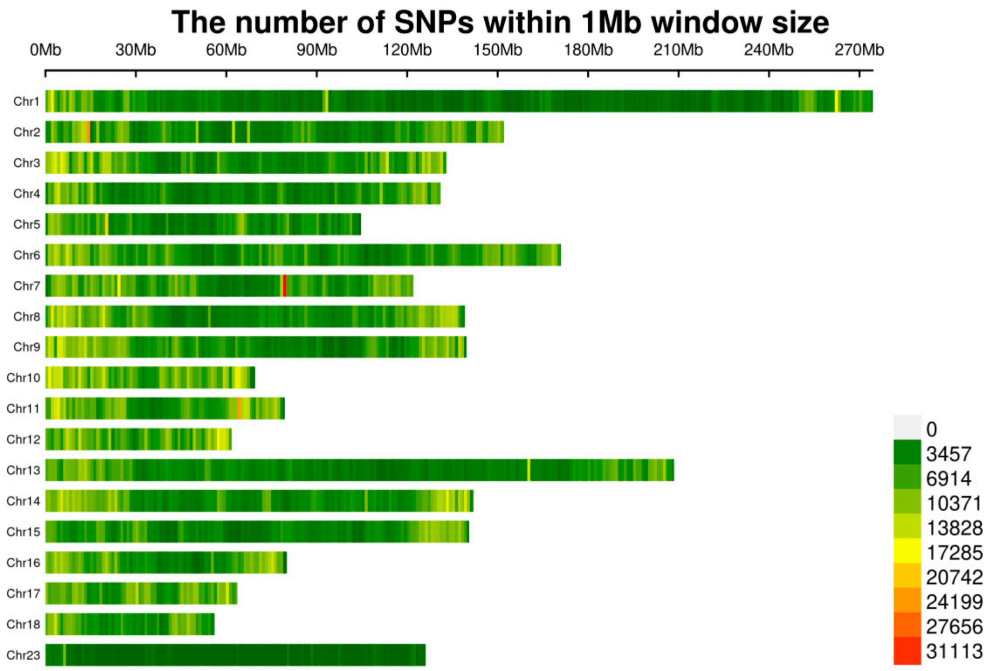

Figure S4. Distribution density of SNPs on each chromosome

[1] Chen S., Zhou Y., Chen Y., Gu J., fastp: an ultra-fast all-in-one FASTQ preprocessor, Bioinformatics. 34 (2018) i884-i890.

[2] Warr A., Affara N., Aken B., Beiki H., Bickhart D.M., Billis K., Chow W., Eory L., Finlayson H.A., Flicek P., Giron C.G., Griffin D.K., Hall R., Hannum G., Hourlier T., Howe K., Hume D.A., Izuogu O., Kim K., Koren S., Liu H., Manchanda N., Martin F.J., Nonneman D.J., O'Connor R.E., Phillippy A.M., Rohrer G.A., Rosen B.D., Rund L.A., Sargent C.A., Schook L.B., Schroeder S.G., Schwartz A.S., Skinner B.M., Talbot R., Tseng E., Tuggle C.K., Watson M., Smith T.P.L., Archibald A.L., An improved pig reference genome sequence to enable pig genetics and genomics research, Gigascience. 9 (2020).

[3] Li H., Aligning sequence reads, clone sequences and assembly contigs with BWA-MEM, arXiv preprint arXiv:1303.3997. (2013).

[4] Pedersen B.S., Quinlan A.R., Mosdepth: quick coverage calculation for genomes and exomes, Bioinformatics. 34 (2018) 867-868.
